# A Web-Based Listening Test System for Cochlear Implant Research and its Validation for Remote Testing

**DOI:** 10.1177/23312165261416179

**Published:** 2026-02-23

**Authors:** Tobias Goehring, Robert P. Carlyon

**Affiliations:** 1 Cambridge Hearing Group, MRC Cognition and Brain Sciences Unit, 2152 University of Cambridge, Cambridge, UK

**Keywords:** cochlear implants, auditory perception, web-based experiments, speech perception

## Abstract

Web-based applications are increasingly used in clinical audiology, driven by the development of mobile, remote technology, and strong demand. Remote applications also have large potential to increase statistical power, accessibility, and diversity in research studies, but their utility and validity are still unclear. We developed and evaluated a web-based listening test system called AUDITO for cochlear implant (CI) research. By exploiting the advances in wireless streaming technology and personal mobile devices, AUDITO can be used to flexibly implement and administer a wide range of listening tests remotely or in-the-lab. The system was designed to be easy to use without programming. Technical features were implemented to ensure signal quality over wireless streaming. A pilot study with 20 experienced CI recipients was performed to evaluate the validity of remote testing across test paradigms. Comparisons of interest included the presentation of stimuli via cable versus Bluetooth streaming and testing remotely versus in-the-lab. Three listening tests were implemented to measure speech perception for sentences, digits in noise and spectro-temporal resolution. A questionnaire was administered to collect user feedback. The system worked reliably with various Bluetooth-compatible setups including desktop and laptop computers, tablets, and smartphones. Test results were consistent between listening modalities and across tested conditions, confirming the validity of web-based testing for these measures. User feedback was positive for system usability and function, while signal quality was not reported to be compromised via streaming. Web-based systems such as AUDITO can facilitate data collection, enable research collaboration and improve accessibility and inclusion in CI research.

## Introduction

Cochlear implants (CIs) compensate for sensorineural hearing loss by directly stimulating the auditory nerve with electrical pulses modulated by sound. Many of the more than 1 million CI recipients worldwide obtain large improvements over their preimplant hearing in terms of speech perception, but there remain limitations for example in noisy listening situations (Boisvert et al., 2020). Many of the listening challenges faced by CI recipients can be attributed to the reduced information transmission at the electrode-nerve interface due to electrical current spread, compromised auditory nerve function (Garcia et al., 2021, 2023) and channel interaction effects (Friesen et al., 2001; Goehring et al., 2020, 2021). Therefore, further research efforts are vital and indeed ongoing to improve CI devices and their outcomes for recipients in future. However, while there has been a lot of progress in various aspects of CI technology, there remain several factors that slow down the advancement (Carlyon & Goehring, 2021).

One key factor that limits CI research is the small number of study participants due to difficulties in recruitment. This results in limited validity, diversity, and representation of the wider population of CI recipients and reduces the statistical power to robustly reveal the effects of interest. A partial solution to this issue would be to combine data across multiple CI research studies to increase statistical power, but this is complicated due to the lack of standardized tests and measurements (Vercammen et al., 2025). Traditionally, CI research studies take place in laboratory settings at research institutes or clinics, with participants attending in person for the duration of the testing and being required to carve out several hours of their day for the traveling and testing time. This in turn means that many adult CI recipients are not able to take part in research studies due to their full-time employment or other commitments. Furthermore, participant reimbursement for travel expenses and the time commitment by researchers for the testing time make CI research studies costly.

Cochlear implant research methods include various tests to assess the perception of different parts of speech such as phonemes, words, or sentences. Speech stimuli may further be mixed with background noise and acoustic simulations of reverberation to investigate CI speech outcomes relevant to real-world listening situations. Research is currently ongoing to improve speech perception by CI recipients, for example, by using computational algorithms that remove background noise from speech to increase its intelligibility, or by processing strategies to improve speech intelligibility directly (Hansen et al., 2020; Koning & Wouters, 2016). Among these methods, deep neural network (DNN) algorithms have recently shown strong promise to increase speech perception for people with hearing loss (Goehring et al., 2016; Healy et al., 2015; Monaghan et al., 2017) and for people that hear with CIs (Gaultier & Goehring, 2024; Goehring et al., 2017; Goehring, Keshavarzi et al., 2019b), but these algorithms have not yet been implemented in CIs. Other important methods within CI research probe the interface between the CI and the auditory nerve so as to derive estimates of spectral and temporal resolution with CI hearing by using nonspeech stimuli (Archer-Boyd et al., 2018; Aronoff & Landsberger, 2013) or to inform novel clinical methods whereby the audiologist deactivates subsets of supposedly ineffective electrodes (Bierer & Litvak, 2016; Garadat et al., 2013; Goehring, Archer-Boyd et al., 2019a; Noble et al., 2014; Zhou, 2017).

In recent years, efforts have been directed toward performing psychoacoustic testing and speech assessment for auditory research using online methods (Peng et al., 2022). Various commercial platforms have become available and popular for participant recruitment and behavioral testing in many areas of research including investigations on hearing. Traditionally, there has been a lot of caution in the auditory research community toward using less-controlled presentation methods due to their lack of accurate calibration and variable sound quality with low-quality speakers or headphones used by the participants. In contrast, recent updates to CI sound processors from all major manufacturers have included wireless sound streaming functionality based on the Bluetooth protocol commonly found in consumer devices such as smartphones, laptops, and tablets. Over the past few years, most CI recipients have now gained access to this technology and use it in their daily life to listen to music or hold telephone conversations via streaming. This facilitates remote testing with CIs, because every participant uses similar hardware and Bluetooth streaming functions, which can easily be replicated in the lab to confirm stimulation patterns.

While early research studies with CI recipients reported differences between listening scenarios including lab-based, office-based and remote settings (Goehring et al., 2012), later studies found no differences in results when using dedicated smart devices with direct audio connection in remote settings (De Graaff et al., 2018). These results have been confirmed by further studies that compared speech tests in the lab against remote testing and found consistent results (Van Wieringen et al., 2021) or even better scores for streamed stimuli presentation than when using loudspeaker presentation (Chen et al., 2021). Other methods that were used for direct comparisons between lab-based and home-based measurements via streaming included tests on the spectro-temporal resolution of CI recipients (Archer-Boyd et al., 2023; Bysouth-Young et al., 2025) and digits-in-noise (DIN) perception (Rootlieb et al., 2024). Remote assessments are now an established part of clinical software suites provided by CI manufacturers that have been used in research studies to investigate longitudinal outcomes (Wasmann et al., 2024), and there is strong demand by CI recipients to consider this technology alongside clinical visits (Sucher et al., 2025). These developments and findings show that remote testing via streaming is a viable and accurate method to assess speech and hearing outcomes with CI recipients. Further development of the remote testing capabilities provided by CI manufacturers is ongoing, but with the important caveat that they only work for their own devices and for specific test setups.

The technology update of CI sound processors with wireless streaming functionality may seem like a standard and iterative step as this technology has long been common in consumer devices. But for CI research and clinical assessments, the availability of a high-quality sound presentation mode similar to lab-based sound presentation has immense potential to facilitate research studies and collaborations, enable more longitudinal interventional studies, increase inclusion and diversity of CI participants in research, and provide possibly substantial cost savings to clinical stakeholders by reducing clinical resources and time commitments for hearing assessments and check-ups.

This motivated the investigation of CI remote testing and the development of an online listening test system called AUDITO, to enable researchers and clinicians alike to utilize remote testing with CI recipients. The combination of wireless streaming, web-based systems, and remote testing in home environments with CI recipients comes with increased complexity for the design choices of a flexible test platform to enable research studies and clinical assessments. Furthermore, early evidence shows that remote testing provides reliable results with CI recipients comparable to in-lab testing, but it is still unclear whether this generalizes to new platforms, device combinations, and test paradigms. To ascertain the feasibility and validity of remote testing for CI research, we present our technical design choices for a testing platform together with the experimental design and evaluation results. We first describe the specific structure and feature of the AUDITO listening test system and then evaluate the outcomes of a validation study to test the system with a group of CI recipients in the lab and remotely across several listening paradigms. The validation study included three listening tests for sentence and digit recognition in noise, a spectro-temporal resolution task, and a questionnaire to gauge experiences and attitudes of the participants. The primary objective of the validation study was to confirm the function and usability of the AUDITO system for remote testing with CI recipients in research studies, with further research questions for comparisons between sound presentation modes, listening settings, and test outcomes within this study and with previous studies to investigate more general aspects of remote testing in CI research.

## AUDITO: A Web-Based Listening Test System

### Overview

**Figure 1.**
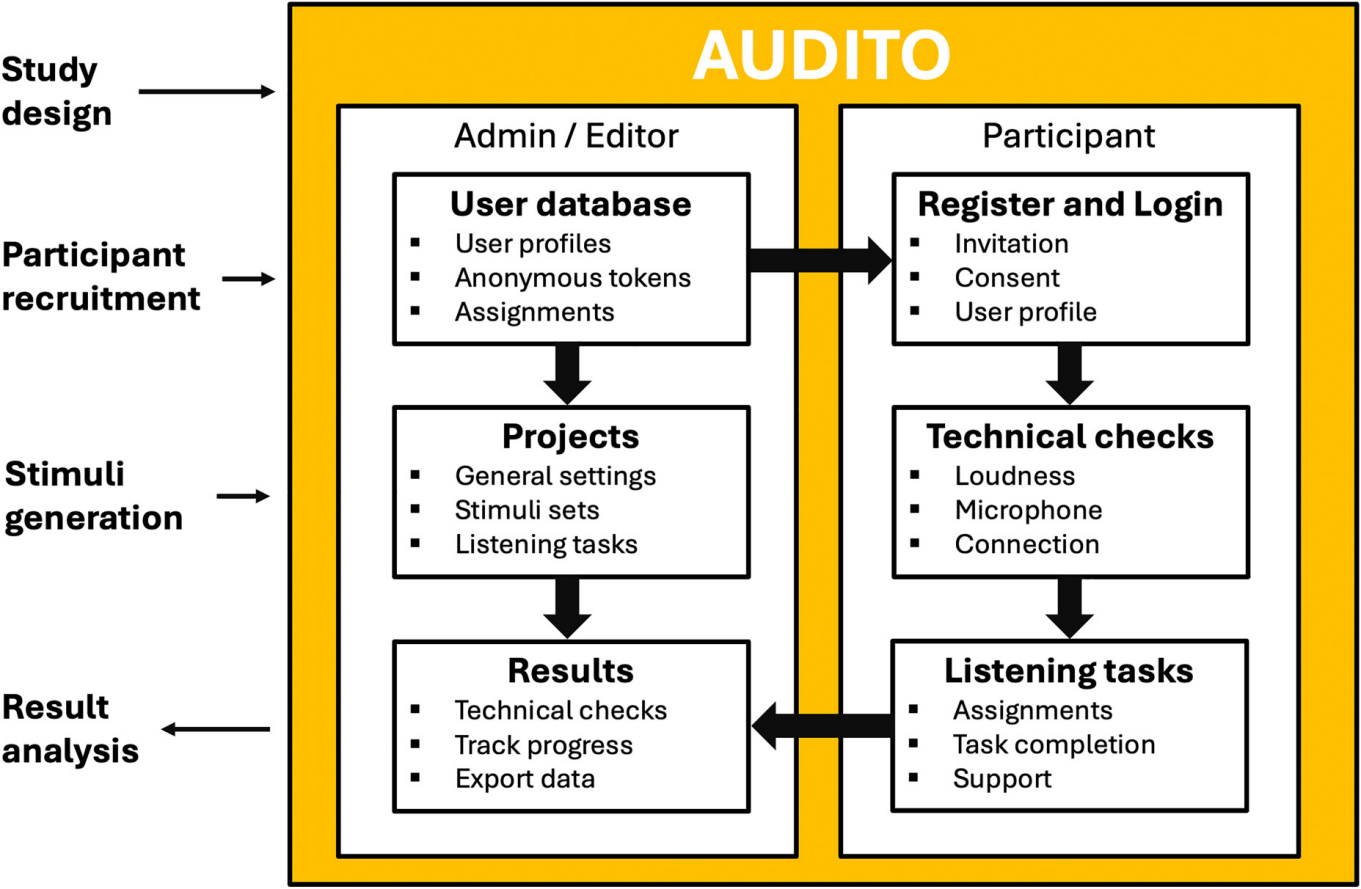
Schematic of the AUDITO listening test system for cochlear implant (CI) research. The system is used to manage users and projects, invite participants, implement listening tasks, and track results. External tasks including the study design, participant recruitment, stimuli generation, and result analysis need to be performed using complementary software or methods.

AUDITO is a web-based listening test system for implementing and performing speech and auditory psychophysics tests either in the lab or remotely. AUDITO was designed for research and clinical studies with CI recipients, but can alternatively be used for headphone testing in acoustic listening experiments or by streaming sounds to a hearing aid. A range of listening tasks can be implemented that cover typical assessments of speech and sound perception as used in research and clinical care with CI recipients. Experimental software for CI research is often based on custom-made MATLAB or Python programs or software toolboxes developed specifically to facilitate CI research (Laneau et al., 2005). While these methods are widely used and provide high degrees of flexibility and experimental control in the lab, they require some programming skills and cannot be run online. The AUDITO system was designed such that listening tests can be quickly implemented and administered online without prior expertise in computer programming; stimuli can, for example, include prerecorded speech sounds and the platform allows the experimenter the flexibility to use computer-generated arbitrary sets of stimuli if required. Project and task management, as well as user and data management, are handled in the web system directly, with result data available in accessible formats for further processing and statistical analysis. A client web application is used online to upload and present test stimuli to the participant and to collect responses via a graphical user interface. Test stimuli are presented for direct streaming via AUX cable or Bluetooth to cochlear-implant devices to ensure high sound quality and experimental control. The AUDITO system runs on a range of web browsers (e.g., Apple Safari or Google Chrome) and devices such as desktop or laptop computers, touch-screen tablets, and smartphones (both Apple and Android devices) with internet connection (Figure 1).

### User Management

AUDITO has three types of users: Participants, Editors, and Admins. All users are first signed up for AUDITO and then assigned to one or more Teams and to a Project. Participants can access the Participant section of the AUDITO system to manage their user profile and run technical checks (see Technical checks and procedures) before starting a listening test and performing the respective listening tasks for the Project they have been assigned to. Participants do not have access rights for any other part of the AUDITO system. Participants can access AUDITO via their user account generated with a personal email address and password or via an anonymous user token generated by an Editor or Admin. Anonymous tokens can be uniquely generated for a specific Project and do not require any personal information, thereby facilitating recruitment and participation in clinical settings. Within the AUDITO system, Participants with user accounts or anonymous tokens are treated identically during the participation in the listening tests of a given Project. Editors can access the Project management section of the system for the specific Project they have been assigned to by an Admin. They can generate and edit listening tasks, generate anonymous user tokens, upload new stimuli sets, and download results data for their assigned Project. Editors do not have access rights beyond their assigned Project. Editors can also access the Participant section of their Project to test the listening tasks in the same way as they would appear for a Participant. Admins have complete access rights to the AUDITO system, specifically the management of existing or new users, generation, and assignments to Team(s) and Projects, in addition to all the capabilities of Editors and Participants.

### Project Management

Listening tests are set up as Projects in AUDITO. Each Project contains one or more *listening tasks* together with the *stimuli sets* used in these tasks and the *result data* for the Participants that were assigned to the Project. The Project is used to specify the order in which the listening tasks appear to the Participants and the number of times each task must be completed (the number of “runs”).

### Listening Tasks

Various listening tests can be implemented in the AUDITO system, including speech perception tests (open- and closed-set phoneme, word or sentence recognition), perceptual ratings, paired-comparison tests, and psychophysical discrimination tests. To implement these tests, several types of listening tasks are available, which differ in their response interfaces and task structure. The response interface options include sets of customizable buttons (up to 5), keypad (digits 0–9), matrix (10 categories for up to 5 key words), text field (written response), and microphone recording (vocal response). These response options allow the creation of listening tasks to measure, for example, a mean opinion score, perceptual detection threshold, recognition accuracy, pitch ranking, and questionnaire or vocal responses. In addition to these main outcome measures, response times are recorded for each response. The task structure of each listening task can be chosen from three options (ordered, paired, or ranked). The ordered task structure is the default setting for presenting a list of stimuli in constant or adaptive mode. The adaptive mode is used to implement adaptive tracking procedures with a staircase method, whereas constant mode plays stimuli in fixed or random order. Adaptive tracking procedures (two-alternative forced choice method) can be defined with various parameters such as step sizes, target response criteria, and number of reversals. The paired-task structure performs a paired comparison for all possible pairs of two stimuli sets and can be used for perceptual ratings or comparisons (Keshavarzi et al., 2018, 2019). The ranked task structure performs the optimally efficient midpoint comparison procedure (Long et al., 2005) to obtain the rank order of a list of audio stimuli as rated by the Participant during paired comparisons. This task uses an optimally efficient method of binary splitting to obtain a unique order of stimuli from paired comparisons, for example, as a tone pitch-ranking procedure. Multiple listening conditions can be used in a listening task in randomized or interleaved order.

Further listening-task parameters specify the stimuli presentation to be automatic or manual, whether stimuli can be repeated or not, feedback settings (overall, item-specific, or none), a maximum number of trials or stimuli to be presented, and instructions to be presented. Listening tasks can be organized into test phases and optionally include a practice phase. Phases can be used to present different test conditions or stimuli during a task. The order of test phases can be fixed or randomized. Each task contains text descriptions before and after the completion of a phase to explain the next listening task or phase to the Participant. Each listening task starts with task-specific technical checks to ensure that the stimuli are presented at a comfortable level and that the technical setup of the Participant is working (see details in “Technical checks and procedures” section). Once a listening task has been completed for the required number of runs, it can be disabled in the Participant interface or made invisible.

### Stimuli Sets

The audio stimuli used by the AUDITO system are organized into stimuli sets and uploaded by the Editor or Admin to a specific Project. Audio stimuli cannot be manipulated within the AUDITO system and must be generated separately using standard audio formats (e.g., .wav and .mp4). Stimuli sets can be shared across Projects to facilitate collaboration and to implement similar listening tasks in several Projects. Each stimuli set consists of the audio stimuli together with a spreadsheet file that describes the stimuli and their properties to the AUDITO system. The stimuli sets are selected during the setup of listening tasks and therefore must match the type and properties of the listening task at hand. The spreadsheet file contains a list with filenames and specific information as required for the listening task response options and adaptive procedures. For example, for an open-set speech perception task, the stimuli set only contains a list of filenames to be used in that task, but for a closed-set speech perception task, the stimuli set also contains information for each audio file regarding the difficulty level, the response options, and the correct answer to enable adaptive tracking procedures. For adaptive procedures, information on the difficulty level allows the procedure to “step through” a list of files in a direction that depends on the correctness of the previous response and using user-specified rules on the size of the steps and for when the procedure should stop. Stimuli sets contain from a few up to several hundreds of audio files, as required for the listening task. For large stimuli sets with many hundreds of audio files, a compressed data format should be used to reduce the amount of data to be downloaded by the user during the test.

### Result Data

The result data are organized for each Project and Participant and can be accessed in various formats to be downloaded and analyzed outside of the AUDITO system. There is an overview of all activities by each Participant that was assigned to the Project to monitor progress and completion of the listening tasks by the experimenter. The result data include data structures with trial-specific information such as stimulus information, participant response and response times, time stamps, as well as the overall settings used in the task. The data can be exported and imported into analysis software to generate data visualizations and perform statistical analysis. Vocal response recordings can be downloaded to be analyzed with software, for example, to perform automatic speech transcriptions or acoustic analysis.

### Technical Checks and Procedures

The AUDITO system incorporates several technical checks and procedures that are used to ensure that a comfortable listening level is used for playback, and that the microphone and streaming connection work appropriately. These methods have been developed and included in the system to achieve high-quality data during the listening tasks. In addition to the technical checks described below, the AUDITO system uses a warning procedure to avoid excessive interruptions during a listening task. Two types of time limits can be defined, whereas the first type issues a warning to the Participant to continue the task after a set duration of inactivity, and the second type terminates the listening task after a set duration of inactivity.

### Loudness Check

The Loudness check plays back a customizable probe stimulus that can be assigned for each Project and asks the Participant to set the loudness to a comfortable level. The probe stimulus should reflect the level and type of the stimuli used during the listening tasks in the Project. All stimuli within a listening task (or Project) should be calibrated before the upload to AUDITO to a consistent level in dB FS and similar to the probe stimulus used for the Loudness check procedure. This ensures that stimuli are presented at a consistent and comfortable loudness during the listening tasks. The Participant should be asked not to change the presentation level of their device after the loudness check has been completed.

### Microphone Check

The Microphone check is used by the Participant to validate that the microphone of their smart device (e.g., smartphone, tablet, or laptop computer) can be used to obtain vocal response recordings. It starts by asking the Participant to take a short recording of their voice with the microphone of the smart device and that is then played back to the Participant to check that the recording has been successful. When starting the Microphone check, the AUDITO system issues a request to the Participant to allow access to the microphone on their device, which is then approved for the rest of the listening session to obtain vocal response recordings. Vocal response recordings are only obtained when the Participant clicks a button to start a recording, and the maximum duration of the recording can be set by the experimenter. Furthermore, the recording button of the AUDITO system incorporates a customizable visual delay (e.g., 500 ms) to ensure that the Participant does not start their vocal response before the recording is active.

### Connection Check

The Connection check allows the Participant to test whether the audio connection is established via Bluetooth streaming when using CIs or hearing aids, or via headphones for participants that hear acoustically. Establishing a direct-audio connection is important for obtaining high-quality data with the AUDITO system, as otherwise the perception of the test stimuli could be confounded by the loudspeaker performance, distance from the smart device, and interfering background noise. A short acoustic measurement is performed to assess whether the stimulus was played back via the loudspeakers of the smart device. Therefore, a short test sound is played back, while a concurrent recording is obtained with the microphone of the smart device. The AUDITO system then performs a comparison calculation between the two signals and determines whether the recorded signal matches the playback signal across several acoustic factors. These factors include the cross-correlation of the two signals in the temporal domain and the correlation of their amplitude spectra, whereby a high correlation value in either of these domains indicates a match between the two signals. If the calculation finds a match between the two signals, then this indicates that the playback was not made using a direct audio connection to the CI (or other device), and the Connection check is therefore failed. Note that this can also be used for other direct audio connections, for example, to check automatically whether headphones are connected without relying on responses from the participant (Milne et al., 2021; Woods et al., 2017). If the calculation determines that the two signals are sufficiently different from each other, the Connection check is passed. This automatic procedure has been optimized to alert the participant if they may not be using a direct-audio connection and to allow further analysis of the result by the experimenter. Acoustic interferences such as background noise or sound leakage from contralateral hearing aids can potentially interfere with the Connection Check and reduce its robustness. The Connection check is therefore currently implemented as a test feature to inform both the participant and experimenter but does not block participation in the listening study. In addition to establishing a valid connection via Bluetooth streaming, a potential limitation of using Bluetooth streaming is the wake-up delay that may cutoff the first part of a stimulus signal after some period of streaming inactivity. This can be avoided in the AUDITO system by specifying a customizable duration of a short silence (e.g., 200 ms) that is appended to the beginning of each stimulus before playback.

### Validation of AUDITO: A Multitask Listening Study with CI Recipients Performed in the lab and Remotely

The goal of this study was to validate the function of the AUDITO listening test system using three listening tests with a group of CI recipients for in-the-lab and online testing. All listening tasks were implemented in the AUDITO system and followed by an online questionnaire to collect experiences with the system. Main factors of interest were the effects of presenting stimuli via direct audio connection versus Bluetooth streaming in the lab, the reliability of test results collected remotely, and to compare results between in-the-lab and remote data collection within this study and against previous studies that used similar tests with CI recipients.

### Participants

In total, 20 English adults (14 female, mean age of 63, ranging from 26 to 80 years) who use a CI to hear took part. Ten participants (5 female) completed the in-the-lab study and 16 listeners (13 female) took part in the online study, with 6 participants (4 female) participating in both study parts. Participants were experienced users of CI devices from three manufacturers (15 Advanced Bionics, 3 Cochlear, 2 MED-EL) with at least 1 year of listening experience with their devices and prior experience with in-person listening tests in clinical or research settings. Most of the participants also had some experience with using Bluetooth streaming with their CI device, for example, to listen to music or holding telephone calls, but a few did not and were provided with documentation on how to establish a Bluetooth connection to their smart device.

### Methods

The three listening tasks implemented in the AUDITO system included a speech-perception task with sentences from a clinically used speech corpus (Bench et al., 1979), a DIN task as commonly used in audiological assessments (Goehring et al., 2025; Smits et al., 2004), and an auditory discrimination task to measure spectro-temporal resolution (Archer-Boyd et al., 2023). This was followed by a questionnaire to gauge participants’ experiences with the AUDITO test system and their attitudes toward remote applications for audiology in general.

### Task I: Speech Perception of BKB Sentences

Sentences from the Bamford-Kowal-Bench (BKB) corpus spoken by a male British English speaker were presented in three processing conditions. The participants were asked to listen to a sentence in each trial and to repeat verbally what they heard while recording their response. The audio recording of the vocal response was performed by the participants themselves using the AUDITO recording feature which displays a button to start and stop the response recording in each trial. The participants completed 15 trials per processing condition in random order, each using one list of the BKB corpus presented in random order. The trials were subsequently scored manually by an experimenter based on the three keywords in each sentence, with a score from 0 to 3 per trial and averaging the scores across trials to obtain a percentage correct score for each list. The processing conditions included the original sentences (“CLEAN”), the sentences mixed with a multitalker babble noise at a signal-to-noise ratio of 5 dB (“NOISY”), and the sentences mixed with noise and subsequently processed with a noise-reduction algorithm based on a DNN (“PROC”) that was trained to remove background noise and retain the dominant speaker in the mix (Thoidis & Goehring 2024). The three processing conditions were chosen to cover varying degrees of difficulty and to reflect speech perception as it may occur in realistic settings for people with CIs. All sentences presented during this task were calibrated to the same root mean square (RMS) level of −25 dB FS to ensure a consistent and comfortable presentation level similar to the probe stimulus used in the Loudness check.

### Task II: DIN Test

Sequences of three-digit stimuli spoken by a male British English speaker were mixed with speech-shaped stationary background noise and presented to the participants via the AUDITO system. The DIN task followed a published procedure (Goehring et al., 2025; Potgieter et al., 2016; Smits et al., 2004). Starting at 0 dB, the signal-to-noise ratio of the stimuli was altered between −20 and 20 dB depending on the response of the participant and following an adaptive 1-up-1-down procedure with a step size of 2 dB. In total, 24 trials were presented and the participant used a virtual keypad to select the digits they heard in each trial, with a response counted as correct if all three digits were identified correctly and in order. An estimate of the speech reception threshold (SRT) was obtained by discarding the first 8 trials and calculating the average SNR of the final 16 trials. All DIN stimuli were calibrated to −25 dB FS to ensure a consistent and comfortable presentation level similar to the probe stimulus used in the Loudness check.

### Task III: Spectro-Temporal Resolution with the STRIPES Test

Spectro-temporal resolution was measured behaviorally using the STRIPES task (Archer-Boyd et al., 2018, 2020) with settings as proposed for online testing (Archer-Boyd et al., 2023). Stimuli consist of concurrent frequency-swept sinusoids and, in every trial, sequences of three stimuli are presented with two of these using downward sweeps and one, the target interval, using an upward sweep. The participant then chose which of the three stimuli were the target interval, which was always either the first or the last interval. A 2-up-1-down adaptive procedure is used to measure the density threshold at which the participant can just perform this task of discriminating upward from downward sweeps (or “stripes”) in the spectro-temporal domain. The stimuli start at a density of 1.1 and adapt with a step size of 0.5 for the first four reversals before changing to a step size of 0.2 for the final four reversals for a total of eight reversals. For each run of the STRIPES task, an estimate of the perceptual threshold was calculated across the final four reversals by averaging the densities. The STRIPES task was run twice, and the final score calculated as the average perceptual thresholds of the two runs. All STRIPES stimuli were calibrated to −25 dB FS to ensure a consistent and comfortable presentation level similar to the probe stimulus used in the Loudness check.

### Questionnaire: Experiences with AUDITO and Attitudes Toward Remote Listening Tests

A questionnaire was performed by the participants a few days after they had completed the listening tasks to assess their experiences, share potential difficulties with the AUDITO system, and collect feedback. This was administered via an online form (Microsoft Forms) and not via the AUDITO system; however, AUDITO has since been updated and can now also be used to administer questionnaires. The questionnaire consisted of 10 questions with answers given using a 7-point scale (from “1—strongly disagree” to “4—neither agree nor disagree” to “7—strongly agree”). The first four questions asked about the usability, readability, and clarity of the AUDITO sign-up process, the supporting document describing the system with a few examples, the AUDITO user interface and the AUDITO website. The next three questions assessed the technical functioning of the listening tasks and the instructions for performing the Connection Check and Microphone Check features. The final three questions asked for participants’ attitudes toward using AUDITO in future, whether they would prefer using AUDITO for research studies and to check their hearing rather than visiting in person. An additional text field allowed participants to make personal comments to highlight any further issues or add feedback in written form.

## Experimental Setup

### Remote Testing

All three tasks—speech perception, DIN, and auditory discrimination—were performed at home remotely. Participants used their own CI sound processor and internet-enabled device (e.g., smartphone, tablet, or laptop computer) to connect to the AUDITO listening test system. The participants were emailed a PDF document that explained the main purpose of the system, included instructions on how to perform the sign-up process and provided step-by-step examples for the listening tasks with screenshots. The participants were asked to set up a Bluetooth connection to their device and perform the AUDITO technical checks and procedures as part of the testing protocol. First, the Connection check was performed by the participant to ensure an active Bluetooth streaming connection between the CI sound processor and the device running the AUDITO system. Second, the Microphone check was performed to ensure that the microphone of the participant's device worked. Third, and at the beginning of each task, the participant was asked to set the presentation level to a comfortable level using a short speech stimulus calibrated to the same RMS level as the test data. The presentation level was then kept constant throughout the testing phase for each task. For the speech task, the remote testing was performed twice in the session so as to measure test–retest reliability in remote settings.

### Laboratory Testing

In addition to remote testing, the speech perception task (only) was also completed for Advanced Bionics CI recipients in the laboratory setting in two presentation modalities, either directly via an audio cable (“DIRECT”) or streamed via Bluetooth ('STREAM”). The stimuli were presented with a CI research processor that was programmed with the clinical settings of each participant. The audio stimuli were played from a laptop computer (Dell XPS) running the AUDITO listening test system in a web browser (Google Chrome). For the DIRECT presentation mode, an Advanced Bionics^®^ Harmony (Valencia, CA) sound processor was used and connected to an external soundcard with an audio cable (Roland^®^ UA-55 Quad-Capture USB, Hamamatsu, Japan). For the STREAM presentation, an Advanced Bionics^®^ Naida sound processor was used and connected via Bluetooth to the laptop. Any microphone input from the sound processor was muted during the experiment. All other settings were identical for the two presentation modalities, and the research sound processors were programmed with the most recent clinical settings of each participant. Front-end sound preprocessing methods such as noise reduction or automatic gain control were deactivated.

## Results

The results were analyzed using the statistical software packages Statsmodels (version 0.14.4) and Pingouin (version 0.5.5) in Python (version 3.12.2) and SPSS (version 28). The data were analyzed using repeated-measures ANOVA (RM-ANOVA) for comparisons with balanced groups (i.e., for each listening setting). For mixed groups (i.e., across listening setting), the data were analyzed using ANOVA with a random factor for participants added.

### Comparison of Speech Perception Testing in the lab and Remotely

**Figure 2.**
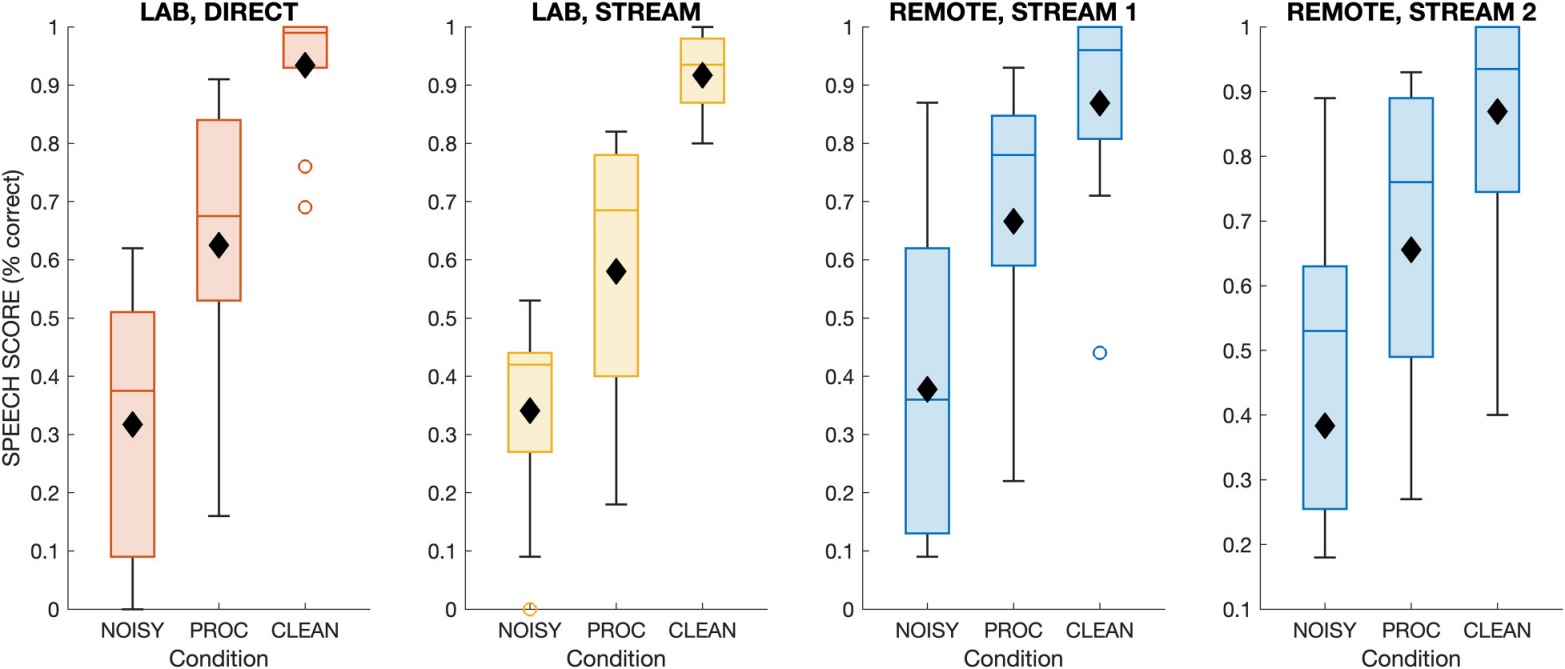
Results for speech perception of BKB sentences for all participants and across listening setting (LAB or REMOTE), modality (DIRECT or STREAM), and processing conditions (NOISY, PROC, CLEAN). Mean scores are shown with black filled markers.

As shown in Figure 2, the speech perception scores with BKB sentences (Task I) exhibited a clear pattern of increasing intelligibility across processing conditions. Overall, group average scores were 38% for NOISY, 64% for PROC, and 90% for CLEAN. This pattern was similar across the four listening settings shown in Figure 2, with average scores ranging from 32% to 48% for NOISY, 58% to 69% for PROC, and 85% to 93% for CLEAN. There was substantial variability across participants, with individual scores ranging from 0% to 89% in NOISY, 16% to 93% in PROC, and 40% to 100% in CLEAN. When combining results from all four listening settings, participants who performed better in one processing condition also tended to perform better in the other processing conditions as shown by positive and statistically significant correlations across participants between NOISY and PROC (*r* = 0.71, *p* < .001), NOISY and CLEAN (*r* = 0.49, *p* = .001), as well as PROC and CLEAN (*r* = 0.59, *p* < .001).

For the LAB results, there was no apparent effect of presentation modality, namely the comparison between DIRECT and STREAM (see Figure 2a and b), which had comparable median scores and overlapping interquartile ranges between conditions. Correlations across participants for individual processing conditions between presentation modalities increased from NOISY (*r* = 0.6, *p* = .063) over PROC (*r* = 0.74, *p* = .014) to NOISY (*r* = 0.83, *p* = .003). Intraclass correlation was also high with a significant coefficient of 0.97 between DIRECT and STREAM [*F*_(9, 10)_ = 68.55, *p* < .001].

Statistical analysis was performed using RM-ANOVA and within-subject factors of presentation modality (DIRECT, STREAM) and processing condition (NOISY, PROC, CLEAN). This revealed a significant main effect of processing condition [*F*_(2, 18)_ = 138.40, *p* < .001], indicating substantial differences in intelligibility across processing types. There was no effect of presentation modality [*F*_(1, 9)_ = 0.99, *p* = .345] nor an interaction effect [*F*_(2, 18)_ = 0.36, *p* = .700]. These findings were corroborated by a Linear Mixed Model (LMM) analysis, which similarly showed significant effects of processing condition (NOISY: β = −0.617, *p* < .001; PROC: β = −0.309, *p* < .001) but no significant effect of listening condition or interaction effects. Post hoc tests using Bonferroni correction revealed significant differences between CLEAN and NOISY (diff = 0.6, *p* < .001), CLEAN and PROC (diff = 0.32, *p* < .001), as well as NOISY and PROC (diff = 0.27, *p* = .001).

For the REMOTE results, there was no apparent difference between the first and second test runs (REMOTE1 and REMOTE2) and a similar overall pattern as for the LAB results (see Figure 2), with comparable median scores and interquartile ranges. Scores were positively correlated between test runs for NOISY (*r* = 0.91, *p* < .001), PROC (*r* = 0.8, *p* = .003), and CLEAN (*r* = 0.93, *p* < .001). Intraclass correlation was also high with a significant coefficient of 0.94 between REMOTE1 and REMOTE2 [*F*_(11, 12)_ = 34.45, *p* < .001]. Bland–Altman analysis showed that the differences between the first and second run fell within +/−1 standard deviation (apart from 1 data point in the NOISY condition), and there was no significant baseline shift indicating comparable performance between runs. Taken together, this indicates strong test–retest reliability for the two test runs performed remotely.

Statistical analysis was performed using RM-ANOVA and within-subject factors of test run (REMOTE1, REMOTE2) and processing condition (NOISY, PROC, CLEAN). Results showed no significant main effect of test run [*F*_(1, 11)_ = 0.59, *p* = .460], indicating that there was no practice or fatigue effect. However, there was a highly significant main effect of processing condition [*F*_(2, 22)_ = 46.07, *p* < .001]. Furthermore, there was no significant interaction [*F*_(2, 22)_ = 2.35, *p* = .119]. These findings were corroborated by an LMM analysis, which similarly showed significant effects of processing condition (NOISY: β = −0.478, z = −10.642, *p* < .001; PROC: β = −0.193, z = −4.301, *p* < .001) but no significant effect of test run or interaction effects. Post hoc tests using Bonferroni correction revealed significant comparisons between CLEAN and NOISY (diff = 0.43, *p* < .001), CLEAN and PROC (diff = 0.18, *p* = .011), as well as NOISY and PROC (diff = 0.25, *p* < .004).

For the comparison between LAB and REMOTE results, speech scores were analyzed using ANOVA with speech score as dependent variable, fixed effects of test setting (LAB or REMOTE), processing condition (NOISY, PROC, CLEAN) and their interaction, as well as a random effect for participants. This analysis revealed a significant main effect of processing condition [*F*_(2, 69)_ = 22.85, *p* < .001], but no effect of test setting [*F*_(1, 69)_ = 0.09, *p* = .762] nor an interaction of the two [*F*_(2, 69)_ = 0.35, *p* = .7]. Post hoc tests with Bonferroni correction revealed significant differences between CLEAN and NOISY (diff = 0.43, *p* < .001), CLEAN and PROC (diff = 0.18, *p* = .016), as well as NOISY and PROC (diff = 0.25, *p* < .001).

**Figure 3.**
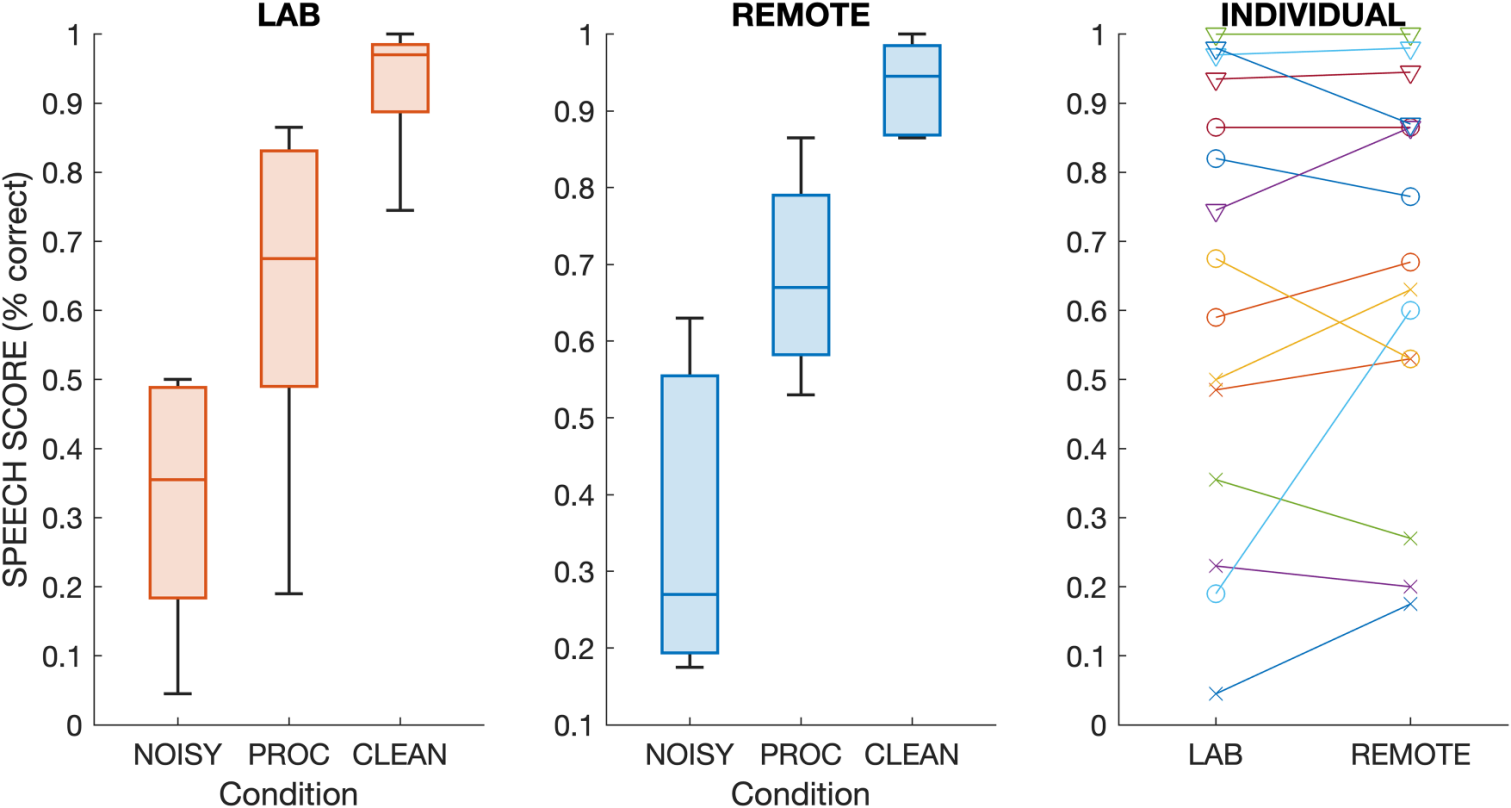
First two panels from left: as for Figure 2 but for the five participants who took part in both listening settings (LAB or REMOTE). Right panel: Individual scores for the two listening settings (cross = NOISY, circle = PROC, diamond = CLEAN).

Results for the five participants who took part in LAB and REMOTE test settings are shown separately in Figure 3. Similar to the overall group results, there were no apparent differences in speech scores between LAB and REMOTE test settings. With one exception, the absolute difference in scores was less than 15% across individuals and processing conditions, with an average absolute difference of 9%.

### Digits-in-Noise Test

**Figure 4.**
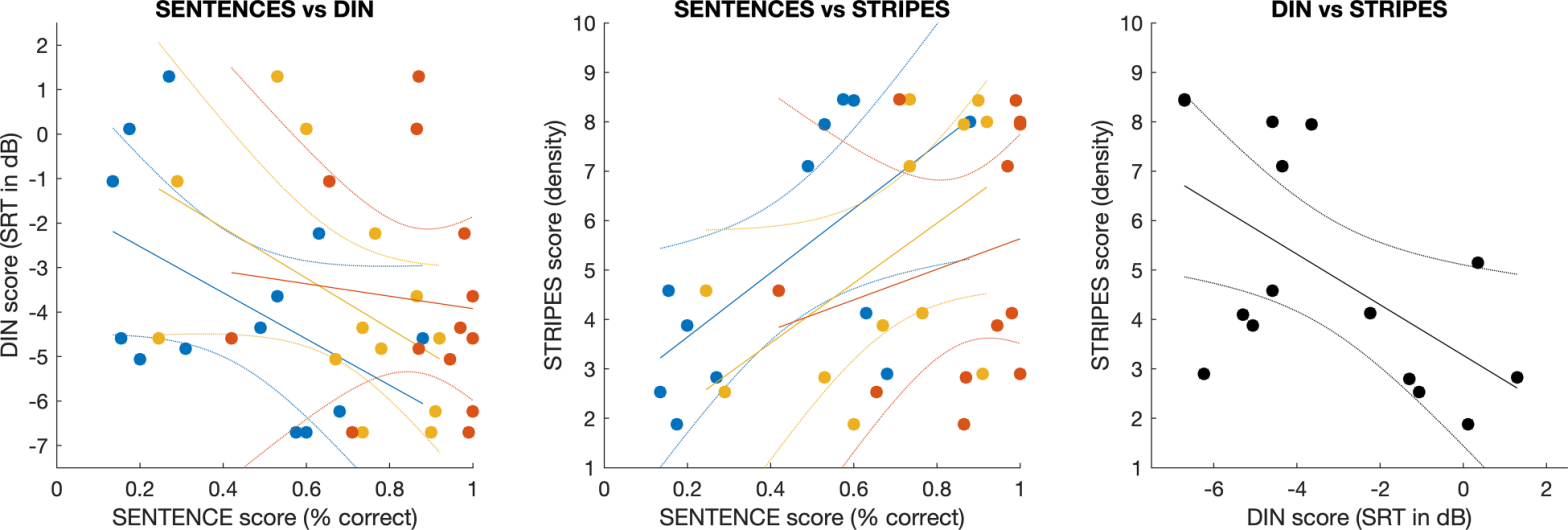
Scatter plots showing correlations between the three listening tasks. Colors indicate processing conditions (blue—NOISY, yellow—PROC, red—CLEAN) with regression lines and 95% confidence intervals (dotted lines) shown for each individually.

The results with the DIN test (Task II) revealed a group average SRT of −3.4 dB (median of −4.5 dB) and ranged from −6.7 dB up to 1.3 dB with a standard deviation of 2.6 dB across participants. The DIN results are shown along with the abscissa in the right-hand panel of Figure 4. Results for participants who completed the DIN test followed a normal distribution (Shapiro–Wilk test: S(16) = 0.916, *p* = .144) with no apparent subgroups and reflecting variability in DIN performance as typically observed with CI recipients. All participants completed the DIN test adaptive procedure with at least 10 reversals, indicating a well-converged estimation of their perceptual thresholds. The DIN test results were comparable to previous studies using the DIN test with CI recipients with mean or median SRTs in a similar range in remote settings (−3.3 dB: Wasmann et al., 2024, −3.8 dB: Van Wieringen et al., 2021) or in the lab (−1.8 dB: Kaandorp et al., 2015). This indicates that DIN test results obtained with the AUDITO system matched previous findings with CI recipients tested online or in the lab.

### STRIPES Test

**Figure 5.**
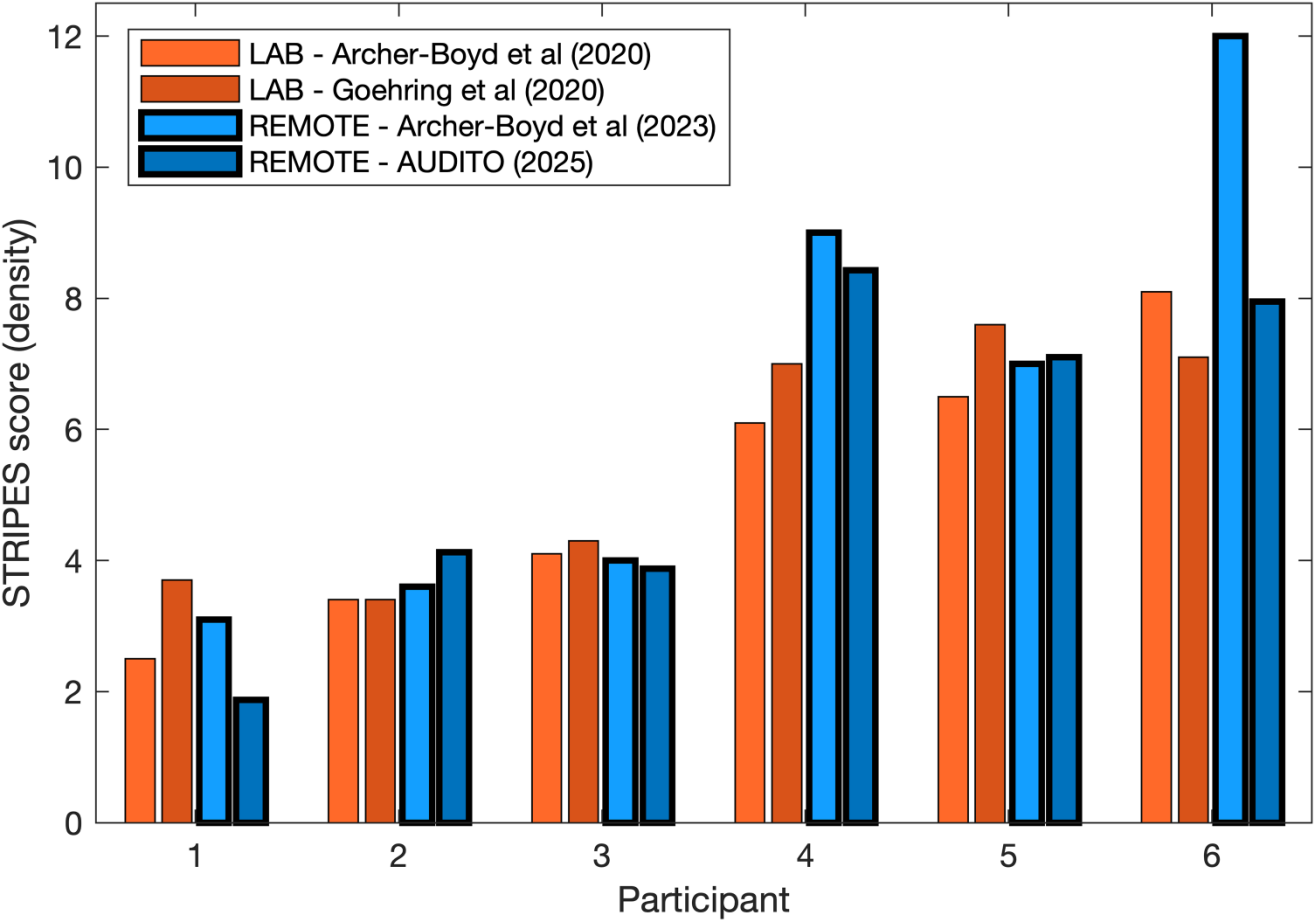
Comparison of STRIPES scores for the six participants that took part in previous studies in the lab and online remotely.

Results for all participants who completed the STRIPES test (Task III) showed a group average density threshold of 5 and ranged from 1.9 up to 8.5 with a standard deviation of 2.4 across participants. The STRIPES results are shown along with the ordinate of the right-hand panel of Figure 4. Scores across participants were distributed evenly but did not strictly follow a normal distribution (Shapiro–Wilk test: S(15) = 0.875, *p* = .041) and reflecting variability in spectro-temporal resolution as typically observed with CI recipients. The two runs of the STRIPES test showed differences from 0.4 up to 1.15 density with an average of 0.5 density (apart from one outlier with a difference of 6.2 between test runs). Figure 5 shows a comparison for a subgroup of six participants who took part in three previous lab-based studies with the STRIPES test. Results for this subgroup were highly consistent between the four studies with a significant intraclass correlation coefficient of 0.82 [*F*_(5, 18)_ = 19.5, *p* < .001] and a 95% confidence interval of [0.54–0.97]. Intraclass correlation was also high for the direct comparison between the two studies using remote testing [ICC = 0.84, *F*_(5, 6)_ = 11.76, *p* = .005] and the two studies using lab testing [ICC = 0.91, *F*_(5, 6)_ = 20.88, *p* < .001]. These intraclass correlation coefficients were within the expected range (95% confidence interval of 0.79–0.98) for the STRIPES test (Bysouth-Young et al., 2025). It again confirms that comparable STRIPES results can be obtained with the AUDITO system as in previous studies using lab-based testing.

### Comparison of Performance Across Listening Tasks

For a comparison, performance scores in the three listening tasks were correlated between BKB sentences and DIN (Figure 4a), between BKB sentences and STRIPES (Figure 4b) and between STRIPES and DIN (Figure 4c) across participants. The speech scores with BKB sentences correlated negatively with the thresholds for the DIN test, but none of these Pearson correlations were statistically significant (NOISY: *r* = −0.49, *p* = .091; PROC: *r* = −0.49, *p* = .089; CLEAN: *r* = −0.09, *p* = .756; all with *N* = 13). There were positive associations between speech perception with BKB sentences and the STRIPES task (*N* = 12), with one correlation statistically significant (NOISY: *r* = 0.63, *p* = .029), while the other two correlations were not statistically significant (PROC: *r* = 0.54, *p* = .069; CLEAN: *r* = 0.22, *p* = .492). There was a negative association between the DIN test and the STRIPES test that was statistically significant (*r* = −0.58, *p* = .025; *N* = 15). When normalizing speech scores in task I for each processing condition, by subtracting the mean performance from the scores in each processing condition, then the correlation across all datapoints in the three processing conditions was significant between speech scores with BKB sentences and the DIN test (*r* = −0.38, *p* = .018, *N* = 39), as well as for the STRIPES test (*r* = 0.48, *p* = .003, *N* = 36).

### Questionnaire: Experiences with AUDITO and Attitudes Toward Remote Applications

**Figure 6.**
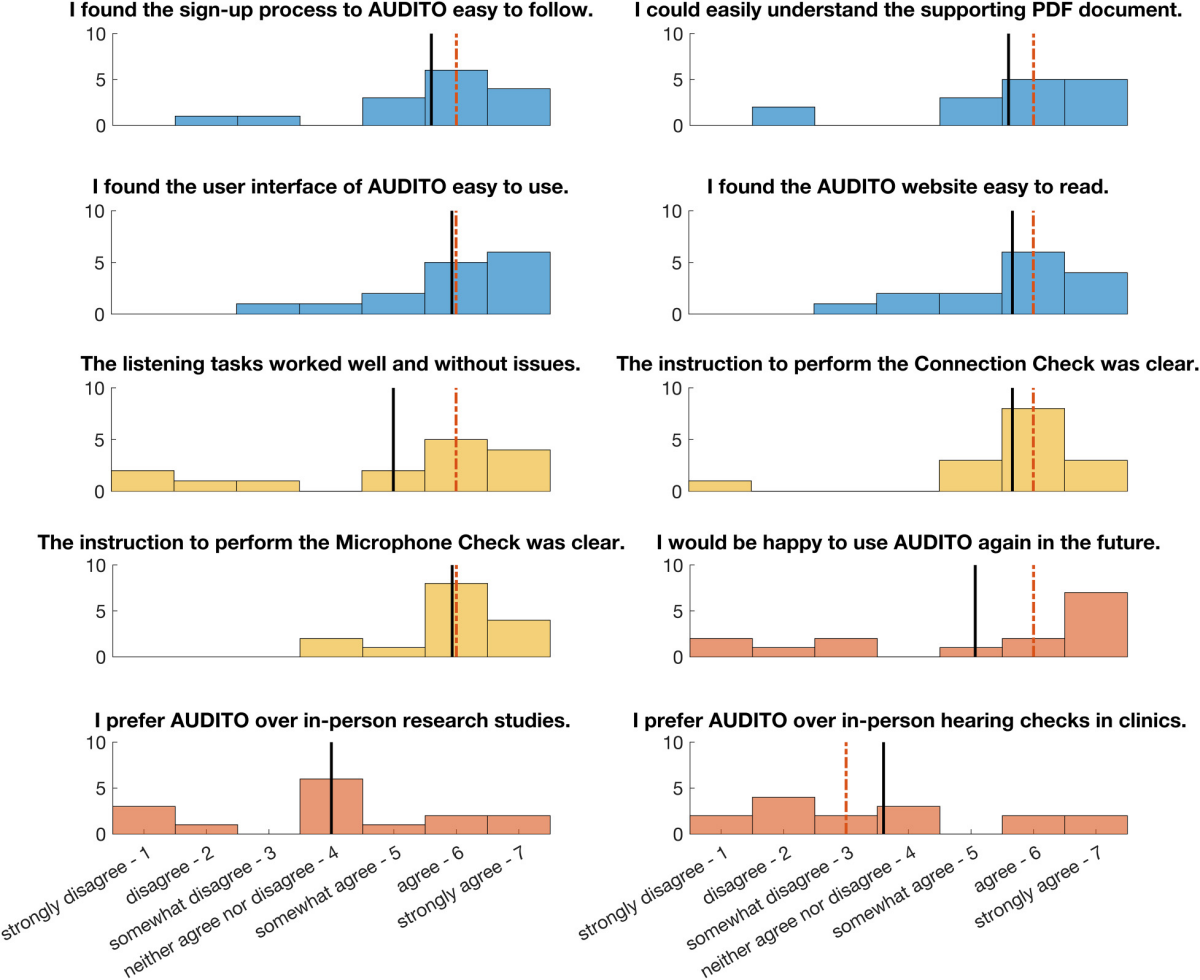
Questionnaire results from the participants who took part in the remote testing with the AUDITO system (*N* = 15). The topics of questions included the user experience with the AUDITO system (Blue), the technical function of the system (Yellow), and attitudes toward remote research and audiology (Red). Mean and median responses are indicated with the black straight and red dotted lines, respectively.

Results from the questionnaire are presented in Figure 6. For the first part of the questionnaire on the usability, readability, and clarity of AUDITO, there was generally good agreement between participants in their responses. Ratings for the sign-up process, supporting document, overall user interface and website readability revealed median ratings of 6 (“agree”) and mean scores from 5.6 to 5.9. Two participants gave lower ratings of 2 (“disagree”) or 3 (“somewhat disagree”) to these questions, showing some room for improvement in the usability of the system and supporting documents. For most participants (13/15), the AUDITO system was easy to use and understand.

The second set of questions concerned the technical function of the AUDITO system, including the performance of the listening tasks and the instructions for the Connection Check and Microphone Check features. Again, the majority of participants gave high ratings for this question, resulting in a median score of 6 and mean score of 5. However, there were some negative responses for the listening task question, where several participants (4/15) experienced technical issues. Based on the text feedback by these participants (last part of the Questionnaire), they reported technical difficulties due to their smartphone or laptop devices and the listening task sounds themselves. For the other questions on the technical checks, there was stronger agreement among participants and ratings were consistently high with median scores of 6 and mean scores of 5.7 and 5.9, respectively. There was one participant for whom the Connection Check did not work on their device for many iterations, but they eventually solved this issue during a lengthy bug fixing process.

The third set of questions gauged the attitudes of the participants on the future use of AUDITO, and their preferences for in-person or remote research studies and clinical sessions for hearing checks. These questions received more mixed responses. Participants were somewhat split on the question whether they were happy to use AUDITO in future, with 5 participants not agreeing and 10 participants agreeing, and median and mean ratings of 6 and 5.1, respectively. There was no clear outcome for group ratings with the next question, where about a third of the participants (4/15) preferred in-person studies over remote studies with AUDITO, a third of the participants (5/15) preferred AUDITO over in-person studies, and the remainder (6/15) was undecided or fine with both options. Lastly, for the final question on preferences regarding clinical in-person visits or remote hearing checks via AUDITO, there was overall some slight disagreement with median and mean ratings of 3 and 3.6, respectively. Four participants stated that they would prefer to complete hearing checks via AUDITO instead of traveling to the clinic in person, but this was not the case for the majority of participants (8/15). A few participants (3/15) did not express a preference for either option. Several participants used the free-text feedback to say that a hybrid approach would be best suited by starting with in-person sessions and then adding follow-up remote sessions.

## Discussion

The AUDITO system was developed to implement, manage, and administer auditory perception tasks online for CI research. It was designed to allow the generation of a variety of listening tests while being easy to use and without the need for any computer programming by the experimenter. The structure of the system facilitates user and project management, such that the task design, settings, and stimuli sets for listening experiments can be shared among researchers. Due to its execution in web browsers, it can be run from any internet-enabled device in the lab or remotely. For experimental control in remote settings, direct audio streaming to the CI sound processor is used to deliver an appropriate sound quality of the stimuli. Several technical features have been implemented in AUDITO to ensure good data quality, including self-administered checks for an active streaming connection, a comfortable listening level, and correct functioning of the device microphone for vocal response recordings. The ease of use and flexibility of the system warrant its application to a variety of use cases across academic and clinical research/practice. A potential clinical application is to allow remote monitoring of changes in the quality of CI hearing, for example, in patients whose hearing fluctuates over time or where the clinician wishes to monitor the success of a change in hardware or clinical programming. For all cases, AUDITO must be supplemented by external stimuli preparation as common with web-based experiment systems and with the advantage of increased flexibility to implement various types of listening tests and assessments with custom-made stimuli. Furthermore, AUDITO saves both summary information and detailed reports of every trial for external analysis, allowing the user to choose the level of data analysis required. By increasing access to listening studies via remote testing and facilitating collaboration across researchers, the diversity and size of participant samples can be increased to improve representativeness, inclusion, and real-world translation of CI research studies (Carlyon & Goehring, 2021).

We validated the AUDITO system in a research study with 20 experienced CI recipients both in the lab and online via remote testing. The web-based test system worked reliably with a variety of listening setups including desktop and laptop computers, tablets, and smartphones with wireless internet and Bluetooth connectivity to the CI sound processor of the participant. The Connection Check feature proved reliable, but some false alarms occurred when higher levels of background noise were present. However, this is not necessarily a disadvantage, as it would indirectly detect unsuitable listening environments due to background noise (Wasmann et al., 2022) and could alert the participant to move to a quieter environment. This group of participants did not report any issues with the sound quality via Bluetooth streaming compared to a direct audio connection or when using the CI microphone; however, it should be noted that for CI recipients with residual hearing in their implanted ear or with hearing in their nonimplanted ear, there could be differences in sound quality with Bluetooth streaming. It is important to note that the AUDITO validation study tested only a limited set of listening tasks and that for other types of tests further validation could be required.

The first task of the validation study measured speech perception scores with the BKB sentences in three listening conditions, including speech mixed with babble noise, after processing with a DNN noise-reduction algorithm (Thoidis & Goehring, 2024), and the clean speech without noise or processing. Speech test results were consistent between test modalities, which included lab-based testing via direct audio connection and Bluetooth streaming. We found similar outcomes and effect sizes with no statistical differences between direct audio and streaming presentation. Similarly, there was no significant difference in speech scores when comparing to remote testing. This was the case even though the remote testing was performed with a partly different group of CI recipients, with five participants taking part in both the lab and the remote testing who showed highly consistent outcomes between test settings (Figure 3). There were highly significant differences in speech scores across processing condition, with recognition scores of about a third correct for the noisy speech, two-thirds correct for the DNN-processed speech and close to perfect recognition for the clean speech. These findings are consistent with previous studies that evaluated DNN noise-reduction algorithms with CI recipients (Gaultier & Goehring, 2024; Goehring et al., 2017; Goehring, Keshavarzi et al., 2019b), which also reported significant speech intelligibility benefits with DNN processing over noisy speech in multitalker background noise, while not fully closing the gap to clean speech. The inclusion of three processing conditions represented a range of listening difficulties to assess the consistency in relative differences across listening setups. The results show that similar conclusions would have been drawn from this experiment whether it was run in the lab—either using direct audio connection or streaming—or performed remotely using streaming.

The second and third tasks of the validation study collected DIN and STRIPES test results as two popular methods used in CI research (Archer-Boyd et al., 2018; Smits et al., 2004). The DIN test results were comparable to previous CI studies (Wasmann et al. 2024; Van Wieringen et al. 2021; Cullington and Aidi 2017; Kaandorp et al. 2015). Some variations in average or median scores across studies are to be expected as most studies employ somewhat different versions of the test (e.g., different voice or noise recordings) and due to the variability in performance across CI recipients leading to a large sampling bias. For the STRIPES test, results were also consistent with the range of performances typically observed for CI recipients tested remotely (Archer-Boyd et al., 2023; Bysouth-Young et al., 2025) or in the lab (Archer-Boyd et al., 2018, 2020; Van Groesen et al., 2023). The same test was used across studies, but again variability across CI recipients and small sample sizes can lead to some variation in group scores due to sampling bias. However, the test reliability of STRIPES was corroborated by a subsample of six participants who took part in several previous studies before (Figure 5). Their STRIPES scores were highly consistent between studies and when compared to the AUDITO validation study, with no apparent procedural learning effect. Intraclass correlations for these six participants were high (ICCs from 0.82 up to 0.9), and within the expected range (95% confidence interval of 0.79–0.98 reported by Bysouth-Young et al., 2025).

For a comparison across participants for the three listening tasks, correlational analyses were performed and showed associations in the expected directions (with better performance on one task predicting better performance on the other tasks). For the speech results using BKB sentences, the correlations did not reach statistical significance for each processing condition individually, probably due to the small number of data points for each. However, when accounting for overall differences between processing conditions and correlating across all data points, the relationships were statistically significant. The relationships were of moderate strength with Pearson's *r* values of 0.38 (BKB sentences vs. DIN), 0.48 (BKB sentences vs. STRIPES), and 0.58 (STRIPES vs. DIN). The somewhat lower association between the BKB sentence test and DIN test found here (when compared to, e.g., Cullington and Aidi 2017; Van Wieringen et al. 2021) can partly be explained by the fact that the former measures an absolute score for a single SNR of 5 dB and is therefore liable to floor effects, whereas the latter measures an adaptive SRT across multiple SNRs and is not affected by floor or ceiling effects. Overall, there were consistent relationships between the three listening tasks, indicating that remote testing via the AUDITO system provided robust results that align with the expected pattern of results as reported in previous studies.

The results from the questionnaire assessing participants’ experiences with the AUDITO system and their attitudes toward remote listening tests showed that overall; there was positive agreement on the ease-of-use, function and clarity of the system and the technical checks. Some participants experienced technical issues that complicated data collection, in particular for vocal response recordings, and can be explained with device compatibility problems. Remote testing can make it more difficult to trouble shoot and help participants to solve technical issues, as most communication is held via email messages. A potential solution would be to first invite participants for an in-person session to explain the function of the system and test its compatibility with the participants’ CI and internet-enabled device. This would preclude inclusion and recruitment of participants who do not wish or indeed cannot travel to attend a research session, but would be suitable, for example, when a patient presents at clinic with hearing through their CI that fluctuates over time. Overall, most participants were able to set up the AUDITO system and streaming to their CI sound processor, as they are already using this feature in daily life. Importantly, responses to the third part of the questionnaire relating to preferences for in-person or remote listening tests and clinical hearing assessments resulted in more mixed answers in this group of CI recipients with prior experience of in-person testing. There was a subgroup of participants who prefer to use remote technologies, while there was also a subgroup of participants who do not wish to use remote listening tests or assessments. Apart from this subgroup, most participants were keen to use AUDITO again in future for research studies, albeit with less enthusiasm for clinical assessments. For clinical assessments, the majority of participants preferred in-person visits to the clinics. As some participants stated, a hybrid approach could work well in future, with the relative proportion of in-person and remote sessions adjusted toward the preferences and requirements for each individual.

In addition to facilitating CI research studies, AUDITO could be helpful for clinical applications and audiological services such as follow-up sessions to assess hearing function longitudinally, or as a supplementary assessment tool for remote fitting sessions to test outcomes remotely pre- and postintervention. A particular advantage of AUDITO is its flexibility, which would allow for specific listening tests as used in clinical practice to be implemented by the clinicians and then performed remotely by their patients. First, pilot studies are currently ongoing to validate the AUDITO system for clinical measurements. This would save time, resources, and cost for clinical stakeholders to free up capacity to be directed toward new CI recipients, or those who may need additional help. Remote testing could be performed at more regular and shorter intervals than in-person testing, thereby increasing sensitivity to potential device faults and enabling faster intervention for unexpected changes in hearing performance. However, essential clinical measurements such as free-field loudspeaker tests that incorporate head-related transfer functions, spatial hearing, and ear-specific cues or the function of the CI microphone would still require in-person assessments. Furthermore, stringent requirements are usually applied to clinical applications, such as for example strict data access rights and security mechanisms as defined in clinical protocols and best clinical practices and these aspects would need to be implemented, certified, and validated for AUDITO before its use in a clinical setting. One step toward meeting these requirements comes from AUDITO's option of inviting participants using anonymized tokens, removing the need for any participant-identifiable data being stored with the results data or in the AUDITO system.

Several other limitations apply to the AUDITO system, among which is the increased responsibility of the participants to use compatible internet-enabled devices capable of Bluetooth streaming, to use the system correctly with these devices, and to perform the listening tasks at an appropriate listening level in a quiet environment without distractions. The AUDITO system does currently not provide a method to perform acoustic calibration to specific sound pressure levels, and this limits its usefulness for listening tasks that require precise absolute stimulus levels or that aim to measure absolute detection thresholds. Remote testing requires careful introduction and clear explanations provided in supporting documents, together with ongoing support for technical issues or potential questions. Email communication is slower than oral conversation, and likely not real time, and can lead to misunderstandings. For research studies, it can be important to reassure CI recipients that their perceived experience during challenging listening tasks (e.g., with adaptive speech-in-noise tests) does not reflect their everyday hearing outcomes and that they may feel as if they were failing the task (inevitable for some trials of an SRT measurement), while performing at a very good level. These types of interactions are more difficult for remote testing, and written communication via email may not provide the same level of explanation, empathy, or clarity as can be provided during in-person sessions. Researchers should take this into consideration and mitigate as best as possible. Another limitation of the AUDITO system is that none of the stimuli can be generated on the fly and need to be composed a priori externally using other software. While this can be a disadvantage for those without the skills to generate and adapt stimuli sets, it means that the system can in principle be used to implement any listening test and is not restricted to a specific selection of stimuli sets. One exception to this are adaptive speech tests that use open-set datasets (e.g., the BKB sentences used in Task I here). To test open-set materials adaptively in real time, one could use written text responses and an automatic grading algorithm, or vocal response recordings together with automatic speech recognition, to obtain response scores. These capabilities are not yet available but could be developed in future to extend the AUDITO system. Both approaches have some potential disadvantages, as text responses on a smartphone screen may be too bothersome for some participants, while automatic recognition systems may produce additional errors for speech recorded in realistic settings with CI recipients, and would then be impossible to disentangle from the speech perception outcomes of the participant.

## Conclusion

These findings support the feasibility and validity of remote testing as an alternative, or addition, to lab-based assessments of auditory perception in CI research studies. Test results with CI recipients were consistent for sentence recognition between listening modalities and settings, showing similar patterns and effect sizes for stimuli presented via cable or streaming as well as between lab-based and remote testing. Results obtained remotely for digits in noise and the STRIPES test were also consistent with previous studies in lab-based and remote settings. The AUDITO system is a new candidate for remote testing that strikes a successful balance between flexibility, to generate a wide range of listening tests, and ease of use, to facilitate accessibility and collaboration. It was rated positively by the participants for its usability and function and can be accessed via anonymous tokens to ensure personal data protection. This platform allows CI researchers to benefit from the advantages of web-based testing for suprathreshold measurements, but there are limitations regarding acoustic level calibration for threshold measurements and access right management for clinical applications. In the meantime, remote test systems such as AUDITO can serve as stand-alone or as hybrid solution in combination with lab-based testing to support and facilitate CI research studies.

## Abbreviations

CI: cochlear implants
DIN: digits-in-noise
DNN: deep neural network
LMM: Linear Mixed Model
RM-ANOVA: repeated-measures ANOVA
SRT: speech reception threshold
